# Pan-enteric Capsule Endoscopy to Characterize Crohn’s Disease Phenotypes and Predict Clinical Outcomes in Children and Adults: The Bomiro Study

**DOI:** 10.1093/ibd/izae052

**Published:** 2024-03-26

**Authors:** Salvatore Oliva, Silvio Veraldi, Giusy Russo, Marina Aloi, Fernando Rizzello, Paolo Gionchetti, Patrizia Alvisi, Flavio Labriola, Maurizio Vecchi, Pini Eidler, Luca Elli, Nikolas Dussias, Gian Eugenio Tontini, Carlo Calabrese

**Affiliations:** Pediatric Gastroenterology and Liver Unit, Department of Maternal Infantile and Urological Sciences, Sapienza University of Rome, Rome, Italy; Pediatric Gastroenterology and Liver Unit, Department of Maternal Infantile and Urological Sciences, Sapienza University of Rome, Rome, Italy; Hepatogastroenterology, Nutrition, Digestive Endoscopy and Liver Transplant Unit, Bambino Gesù Children’s Hospital, IRCCS, Rome, Italy; Pediatric Gastroenterology and Liver Unit, Department of Maternal Infantile and Urological Sciences, Sapienza University of Rome, Rome, Italy; Pediatric Gastroenterology and Liver Unit, Department of Maternal Infantile and Urological Sciences, Sapienza University of Rome, Rome, Italy; IBD Unit, IRCCS, Azienda Ospedaliero-Universitaria di Bologna, Italy; IBD Unit, IRCCS, Azienda Ospedaliero-Universitaria di Bologna, Italy; Alma Mater Studiorum, Università di Bologna, Italy; Pediatric Gastroenterology Unit, Maggiore Hospital, Largo Bartolo Nigrisoli, 2, 40133 Bologna, Italy; Pediatric Gastroenterology Unit, Maggiore Hospital, Largo Bartolo Nigrisoli, 2, 40133 Bologna, Italy; Department of Pathophysiology and Transplantation, University of Milan, Italy; Division of Gastroenterology and Endoscopy, Foundation IRCCS Ca’ Granda Ospedale Maggiore; Division of Gastroenterology and Endoscopy, Foundation IRCCS Ca’ Granda Ospedale Maggiore; Division of Gastroenterology and Endoscopy, Foundation IRCCS Ca’ Granda Ospedale Maggiore; IBD Unit, IRCCS, Azienda Ospedaliero-Universitaria di Bologna, Italy; Department of Pathophysiology and Transplantation, University of Milan, Italy; Division of Gastroenterology and Endoscopy, Foundation IRCCS Ca’ Granda Ospedale Maggiore Policlinico, Milan, Italy; IBD Unit, IRCCS, Azienda Ospedaliero-Universitaria di Bologna, Italy

**Keywords:** pan-enteric capsule, noninvasive endoscopy, monitoring, intestinal inflammation

## Abstract

**Background:**

Pan-enteric capsule endoscopy (PCE) provides useful information for the management of Crohn’s disease (CD), especially in children. No study has evaluated the ability of PCE to characterize CD phenotypes and outcomes in children and adults.

**Methods:**

In a prospective multicenter observational study, we recruited patients with CD >6 years from 4 centers in Italy. Patients underwent clinical, biomarker assessment and PCE. Lesions were graded using the PCE system. For each segment, the most common lesion (MCL), the most severe lesion (MSL), and the extent of involvement were defined. Disease severity, extent, and clinical outcomes were compared between children and adults. A logistic regression analysis was used to identify predictive factors for negative outcomes in both age groups.

**Results:**

One hundred ninety-four consecutive patients (adults/children: 144/50) were evaluated for a total of 249 procedures. Children were more likely to have extensive disease, particularly in the colon. Higher MCL scores were independently associated with treatment escalation (odds ratio [OR], 4.09; 95% CI, 1.80-9.25; *P* = .001), while >30% disease extent was more indicative of clinical and endoscopic relapse (OR, 2.98; 1.26-7.08; *P* = .013). Disease extent was the only factor associated with endoscopic recurrence in children (OR, 4.50; 95% CI, 1.47-13.77; *P* = .008), while severe lesions in adults provided a better predictor of treatment escalation (OR, 4.31; 95% CI, 1.52-12.1; *P* = .006). Postexamination, PCE contributed to a change of therapy in 196/249 (79%) of the procedures.

**Conclusions:**

PCE allowed the characterization of CD phenotypes in children and adults by assessing disease severity and extent, which are of different importance in predicting clinical outcomes in these age groups.

**Key Messages**

**What is already known?**

Capsule endoscopy (CE) is recommended for assessing small bowel inflammation in Crohn’s disease (CD) patients. Pediatric CD often involves the small intestine proximal to the terminal ileum, and a 1-step device to assess the entire digestive tract is preferred, especially in children. The new pan-enteric capsule (PCE) provides an efficient assessment of both the small and large intestine in patients with CD.

**What is new here?**

This study shows that pediatric CD is more pan-enteric and severe in the colon compared with adult CD. Pan-enteric capsule findings have an impact on disease outcomes and therapeutic decisions, with both severity and extent affecting clinical outcomes, especially in children.

**How can this study help patients care?**

The use of PCE can help differentiate disease phenotypes and guide treatment decisions in pediatric and adult patients with CD. Pan-enteric capsule enables more precise monitoring of disease activity and response to treatment, leading to better disease management and outcomes.

## Introduction

Capsule endoscopy (CE) is of particular importance in the diagnosis and monitoring of Crohn’s disease (CD).^1–3^ Therefore, guidelines have recently recommended this tool for small bowel (SB) assessment in both patients with suspected and confirmed CD.^4,5^ Ileocolonoscopy remains the gold standard for evaluating colonic mucosal inflammation, but its invasiveness combined with the need for repeated sedation justifies the validation of less invasive methods.^6^ In addition, ileocolonoscopy is unable to assess the small intestine proximal to the terminal ileum, which is commonly involved in pediatric CD and in a significant proportion of adults.^7–9^ Currently, a complete GI assessment in patients with CD often requires multiple diagnostic investigations including ileocolonoscopy, cross-sectional imaging, and CE.^5^ Therefore, the concept of a 1-step device to assess the entire digestive tract is particularly attractive to monitor disease over time, especially in children, for whom less invasive methods should be preferred.^10–12^ Several studies have described the use of the double-headed colon capsule to assess both the small and large intestine in CD in children and adults.^13–15^ Over the years, the CE system has been updated by developing special software to detect and score inflammatory lesions; and a novel pan-enteric capsule (PCE; PillCam Crohn’s, Medtronic, Dublin, Ireland) has recently been released.^16^ The PCE enables a simultaneous efficient assessment of the small and large intestine. This new model provides a way to localize and grade disease activity and quantify its extent, as well as to compare back-to-back studies to allow assessment of response to therapy. Feasibility studies have shown that PCE is safe and has greater diagnostic yield than ileocolonoscopy even in the terminal ileum and colon.^16–18^ However, the implications for treatment decisions and correlation with biochemical disease markers in adults and children have not yet been studied. In children, PCE has been well tested as a noninvasive tool to assess mucosal healing, which is the most important therapeutic goal as it is associated with better outcomes.^19^ When comparing CD location and severity between children and adults, it is expected that if more comprehensive GI assessments were performed, there would be a greater likelihood of a broader extent of the disease in children.^20,21^ It is known that pediatric patients are more often characterized by inflammatory and pan-enteric disease than adults, who have more stricturing or penetrating disease.^22^ In this prospective, multicenter, observational study (The BOMIRO study), we aimed to evaluate the ability of PCE to characterize disease location and severity throughout the entire GI tract in pediatric and adult patients with CD, thereby characterizing the 2 phenotypes. In addition, we aimed to assess the correlation with biochemical disease markers and the impact of PCE on clinical disease outcomes and treatment decisions.

## Patients and Methods

### Patients

Eligible patients were prospectively and consecutively recruited between 2018 and 2021 at 4 different pediatric and adult tertiary referral centers for IBD. Participating units were Pediatric Gastroenterology and Liver Unit of Sapienza University of Rome, Rome, Italy; Gastroenterology and Endoscopy Unit, Fondazione IRCCS Ca’ Granda Ospedale Maggiore Policlinico, Milan, Italy; Department of Gastroenterology, Sant’Orsola Hospital, Bologna, Italy; and Pediatric Gastroenterology Unit, Pediatric Department, Maggiore Hospital, Bologna, Italy. These centers created a network specifically to collect prospective observational data on PCE use in IBD patients, which has been called “BOMIRO,” according to the initials of the participating cities.

Inclusion criteria included (1) age ≥6 years; new small bowel and/or colonic CD (all pediatric and adult patients at the time of diagnosis); (2) need for endoscopic evaluation with PCE (for staging to assess disease extension even after the diagnosis and/or follow-up in case of unexplained symptoms); and (3) a clinical follow-up after PCE of at least 6 months to assess response to treatment. Exclusion criteria were (1) dysphagia; (2) renal insufficiency; (3) known stricturing or perianal CD; (4) prior abdominal surgery; and (5) diagnosis of ulcerative colitis or IBD-U.

Data collected included age, gender, disease duration and location, medication history (before and after PCE), indication, blood test parameters, fecal calprotectin (FCP), and Pediatric CD Activity Index (PCDAI) or CD Activity Index (CDAI).^23,24^ Blood tests and FCP were included if performed in the month prior to capsule endoscopy and without any changes in treatment. A C-reactive protein (CRP) greater than 5 mg/L and FCP greater than 150 mg/kg were considered elevated. Disease phenotype was reported using the Montreal and Paris classification in adults and children, respectively.^25^ Disease outcomes were also recorded over the follow-up period: need for corticosteroids, treatment escalation, need for surgery or hospitalization related to the disease, occurrence of endoscopic relapse (as detected by PCE and/or ileocolonoscopy within 6- to 24-month period), and clinical relapses, as defined by an increase in CDAI and PCDAI values above the remission threshold (>150 and >10, respectively) or from previous evaluation.

### Pan-Enteric Capsule

Pan-enteric capsule was performed at the discretion of the clinician after consultation with the patient as part of routine practice. Ileocolonoscopy and small bowel radiology or PCE were discussed with patients before considering any instrumental evaluation. Patients who received a PCE were included in the study. Pan-enteric capsule was performed according to individual unit protocols based on local and/or published experience.^19,26^ In adults, a slit-dose polyethylene glycol (PEG) regimen was used for bowel preparation, while in children, a spit-dose low-volume regimen with sodium pycosulfate was preferred for colon cleansing. After the pan-enteric capsule (PCE) exited the stomach and detected the small bowel, as confirmed by real-time viewing, a first booster of oral sodium phosphate solution (20 mL) was given. Three hours later, a second booster of sodium phosphate (10 mL) was administered to promote distal movement of the capsule through the bowel and improve capsule excretion. If necessary, one Bisacodyl suppository (10 mg) was given to facilitate bowel emptying and excretion of PCE 3.5 hours after the second booster. These dosages and regimens were selected to optimize bowel preparation and enhance the excretion of the PCE. To minimize the risk of retention, reliable patency tests were performed in all patients with established Crohn’s disease, in accordance with European guidelines.^5^ These tests included the use of a patency capsule as per routine and cross-sectional imaging in low-risk patients only. The readers were experienced gastroenterologists or pediatric gastroenterologists with extensive experience in PCE and over 200 studies reviewed in the past 2 years. The PillCam Crohn’s platform includes a software reporting system that divides the gastrointestinal tract into small bowel tertiles (SB1, 2 and 3: by time and speed of capsule transit) and colon, so that inflammation in each region can be graded from 1 to 3 (none, mild, moderate, severe) depending on size and depth of ulcers (Supplementary Table 1). The reader records the most common lesion (MCL) and most severe lesion (MSL) in each region and the extent (in percentage: 0%; 0%-10%; 10%-30%; 30%-60%; 60%-100%) of the region involved, including the colon. Active disease was defined as the presence of mild ulceration (MCL 1) affecting at least 10% of the segment or the presence of moderate or severe ulceration (MSL 2 or 3) of any extent. A comprehensive formal report was prepared for referring clinicians who had sole responsibility for therapeutic interventions. In addition, the Lewis score was also calculated in the SB.^27^

The definition of medical treatment adjustment after evidence of inflammation was based on the introduction of corticosteroids or enteral nutrition; the introduction or optimization of immunosuppressants (methotrexate or thiopurines); the introduction and optimization of biologics (increase of dosage or reduction of time intervals); or the introduction of both immunosuppressants and biologics. Similarly, a reduction in treatment intensity was feasible in the event of achieving endoscopic remission.

### Study Design and Objectives

This multicenter, prospective, observational study in consecutive patients with suspected and established CD aimed (1) to examine and compare the disease phenotype (severity and extent) as assessed by PCE in children and adults; (2) to compare clinical and biochemical markers with PCE results and assess their value in detecting significant intestinal inflammation; and (3) to assess the impact of PCE findings on clinical disease outcomes and therapeutic management over the follow-up period in the entire cohort, as well as in both age groups.

Participating investigators designed, approved, and analyzed the study protocol. Patients were seen as part of routine clinical practice. Pan-enteric capsule was conducted either at the time of diagnosis or during follow-up visits, which included assessments for clinical relapse or treatment response evaluation following 6 months of therapy modification. The specific timing and frequency of PCE examinations adhered to the local practice protocols at each participating site. A specific and dedicated online platform was created to collect data from all participating units. All identifiable medical information has been removed, and all analyses were performed using anonymized data. The data underlying this article are available in a RedCap Database (https://redcap.unibo.it/malattieinfiammatoriecronicheintestinali/) and can be accessed with a unique identifier. The study protocol was defined in accordance with the Declaration of Helsinki and approved by the local ethical committees. All authors reviewed the study data and approved the final manuscript.

### Statistical Analysis

The Kolmogorov-Smirnov test was performed to check the data distribution. Continuous variables were expressed as medians and interquartile range (25th-75th percentiles) or mean ± standard deviation, as appropriate. Comparison of continuous variables between 2 groups was performed using the Mann-Whitney or *t* test. Categorical data were expressed as proportions, and the χ^2^ or Fisher tests were used for the comparisons. Spearman’s rank correlation analysis was performed to assess the concordance between Lewis score and endoscopic features. To evaluate the association between endoscopic findings (independent variables) and disease outcomes (dependent variables), we conducted both univariate and multivariate logistic regression analyses. The variables selected for inclusion in the multivariate models were based on their clinical relevance and included covariates such as gender and disease location. This approach allowed us to assess the independent impact of endoscopic findings on disease outcomes while controlling for potential confounding factors. Moreover, the most inflamed segment among those assessed by the capsule was selected to determine the values for MSL, MCL, and disease extension. When multiple segments were affected, we gave priority to the segment displaying more severe inflammation and/or extensive disease. A *P* value <0.05 was considered significant. Statistical analysis was performed with SPSS v.22.0 (IBM Corp., Armonk, New York, United States).

## Results

### Patient Population

**Figure 1.**
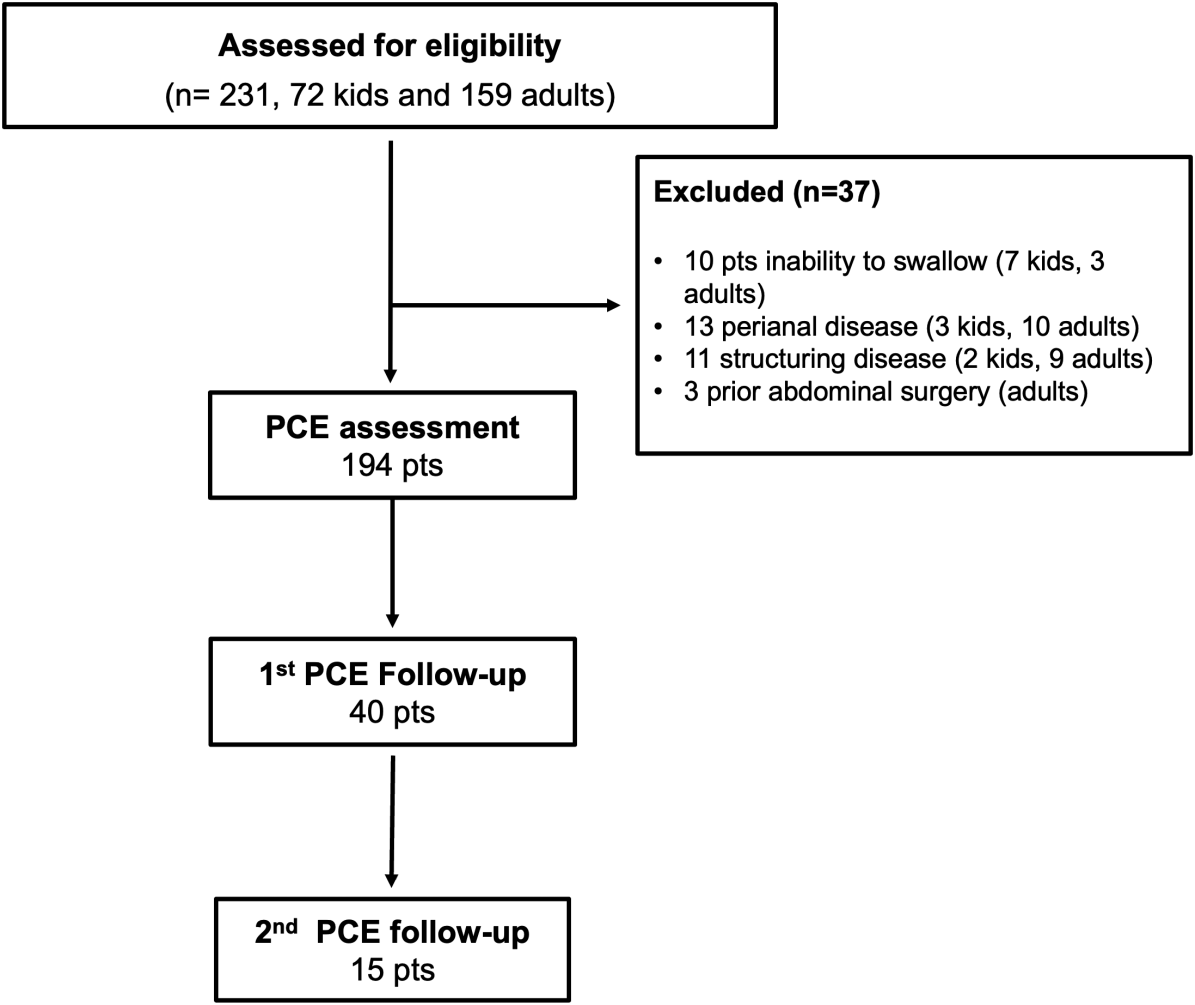
Study flowchart with number of enrolled patients.

Out of the 231 patients assessed for eligibility, a total of 194 patients with CD were recruited, including 50 children (26%) and 144 adults (74%). All patients received 1 or more PCE for a total of 249 procedures analyzed (90 in children and 159 in adults; 194 as baseline assessment, 40 and 15 as first and second PCE follow-up, respectively; Figure 1). The clinical and demographic characteristics of pediatric and adult patients are described in Table 1.

**Table 1.** General characteristics of the adult and pediatric population (no. patients 194, no. procedures: 249)

		Adult		Pediatric	
Sex (M/F)		77/67 (53.5%/46.5%)		35/15 (70%/30%)	
Age (years)		36.3 (25.1-49.6)		14.1 (12.1-16.3)	
Duration of follow-up (months)		40 (12.0 -56)		20 (11-34)	
Disease duration (months)		52 (11-126)		44 (6-83)	
**Adult**	**Pediatric**				
CDAI	PCDAI (median, IQR)	173 (97-248)		20.00 (5-35)	
CDAI < 150	PCDAI < 10	48 (30%)		28 (31.1%)	
CDAI 151-219	PCDAI 10-30	46 (29%)		29 (32.2%)	
CDAI 220-450	PCDAI 30-45	23 (14.5%)		13 (14.5%)	
CDAI > 450	PCDAI > 45	24 (15%)		13 (14.5%)	
N/A	N/A	18 (11.3%)		7 (7.7%)	
Hemoglobin (g/dL) (n = 190, 76.3%)		13.05 (11.8-13.9)		13.1 (12.1-14.4)	
C-reactive protein (mg/dL) (n = 211, 84.7%)		0.9 (0.50-2.63)		0.37 (0.09-2.38)	
Fecal calprotectin (μg/g) (n = 187, 75%)		242 (44-947)		243 (51-560)	
Indications for PCE (n, %)		159 (64%)		90 (36%)	
-- New diagnosis/disease extension		58 (36%)		43 (47%)	
- Response to treatment		35 (22%)		20 (22%)	
-- Disease Recurrence		66 (42%)		27 (30%)	
**Therapy**		**Before PCE**	**After PCE**	**Before PCE**	**After PCE**
Immunomodulators		27 (17%)	9 (5.7%)	11 (12.2%)	13 (14.4%)
Glucocorticoids		13 (14.5%)	10 (6.3%)	10 (11.1%)	10 (11.1%)
Antibiotics		6 (3.8%)	1 (0.6%)	6 (6.7%)	8 (8.9%)
5-Aminosalicilates		31 (19.5%)	18 (11.3%)	13 (14.4%)	12 (13.3%)
Methothrexate		0 (0%)	1 (0.6%)	6 (6.7%)	5 (5.6%)
Anti-TNF-α		24 (15%)	65 (40.8%)	40 (44.5%)	45 (52.3%)
Vedolizumab		4 (2.5%)	12 (7.5%)	1 (1.1%)	1 (1.1%)
Ustekinumab		6 (3.8%)	14 (8.8%)	1 (1.1%)	3 (3.3%)
Enteral nutrition		0 (0%)	0 (0%)	4 (4.4%)	13 (14.4%)
Others		1 (0.6%)	1 (0.6%)	10 (11.1%)	6 (6.7%)
**Localization** (number)					
L1		50 (34%)		17 (34%)	
L2		21 (16%)		5 (10%)	
L3		45 (31%)		11 (22%)	
L4		28 (19%)		17 (34%)	

### Disease Phenotypes

Endoscopic lesions detected by PCE in the total cohort, pediatric group, and adult group are listed in Table 2. Both severity and extent were reported in each GI segment (SB1, 2, 3, and colon) using capsule platform classification with MSL, MCL, and the disease extent. No lesions were found in 22 (8.8%) examinations; therefore, these subjects were classified as inactive.

**Table 2.** Endoscopic features of the population (249 procedures: 159 in adults, 90 in children).

	SB First segment				SB Second segment				SB Third segment				Colon			
	Overall	Adults	Pediatrics	*P*	Overall	Adults	Pediatrics	*P*	Overall	Adults	Pediatrics	*P*	Overall	Adults	Pediatrics	*P*
**Most Severe Lesion**																
None-Mild	76.3%	76.7%	75.6%	0.877	79.9%	83%	73.5%	0.138	50.6%	54.4%	43.3%	0.088	73.9%	74.1%	73.3%	0.027
Moderate-Severe	23.7%	23.3%	24.4%		20.1%	17%	26.5%		49.4%	45.6%	56.7%		12.4%	8.9%	18.8%	
N/A													13.7%	17%	7.9%	
**Most Common Lesion**																
None-Mild	86.8%	87.4%	85.4%	0.700	87.2%	89.3%	83%	0.236	60.6%	63.9%	54.6%	0.223	78.3%	77.3%	79.9%	0.053
Moderate-Severe	13.2%	12.6%	14.6%		12.8%	10.7%	17%		39.4%	36.1%	45.4%		8%	5.7%	12.4%	
N/A													13.7%	17%	7.7%	
**Disease Extent**																
0%	40.6%	37.7%	45.6%	<0.001	61%	66.7%	51.1%	<0.001	30.5%	34.6%	23.3%	<0.001	58.2%	62.9%	50%	0.005
0-10%	41%	52.8%	20%		22.1%	27%	13.3%		39%	43.4%	31.1%		16.9%	13.8%	22.2%	
10-30%	9.6%	8.8%	11.1%		6.4%	4.4%	10%		14.5%	15.7%	12.2%		6%	3.1%	11.1%	
30-60%	3.2%	0.6%	7.8%		4.8%	1.3%	11.1%		7.2%	5%	11.1%		1.6%	1.3%	2.2%	
60-100%	4.4%	0%	12.2%		3.6%	0%	10%		6.8%	0.6%	17.8%		2.8%	1.3%	5.6%	
N/A	1.2%		3.3%		2%	0.6%	4.4%		2%	0.6%	4.4%		14.5%	17.6%	8.9%	

Abbreviations: SB, Small bowel.

A statistically significant difference was observed between pediatric and adult CD in disease extent, as children were more frequently affected by extensive disease in each GI segment than adults (*P* < .05), particularly in the colon where higher MSL and/or MCL scores were detected (*P* < .05). Therefore, CD in pediatric patients of this cohort was more extensive and more severely affecting the colon.

### Comparison With Demographics, Clinical Scores, and Biomarkers

**Figure 2.**
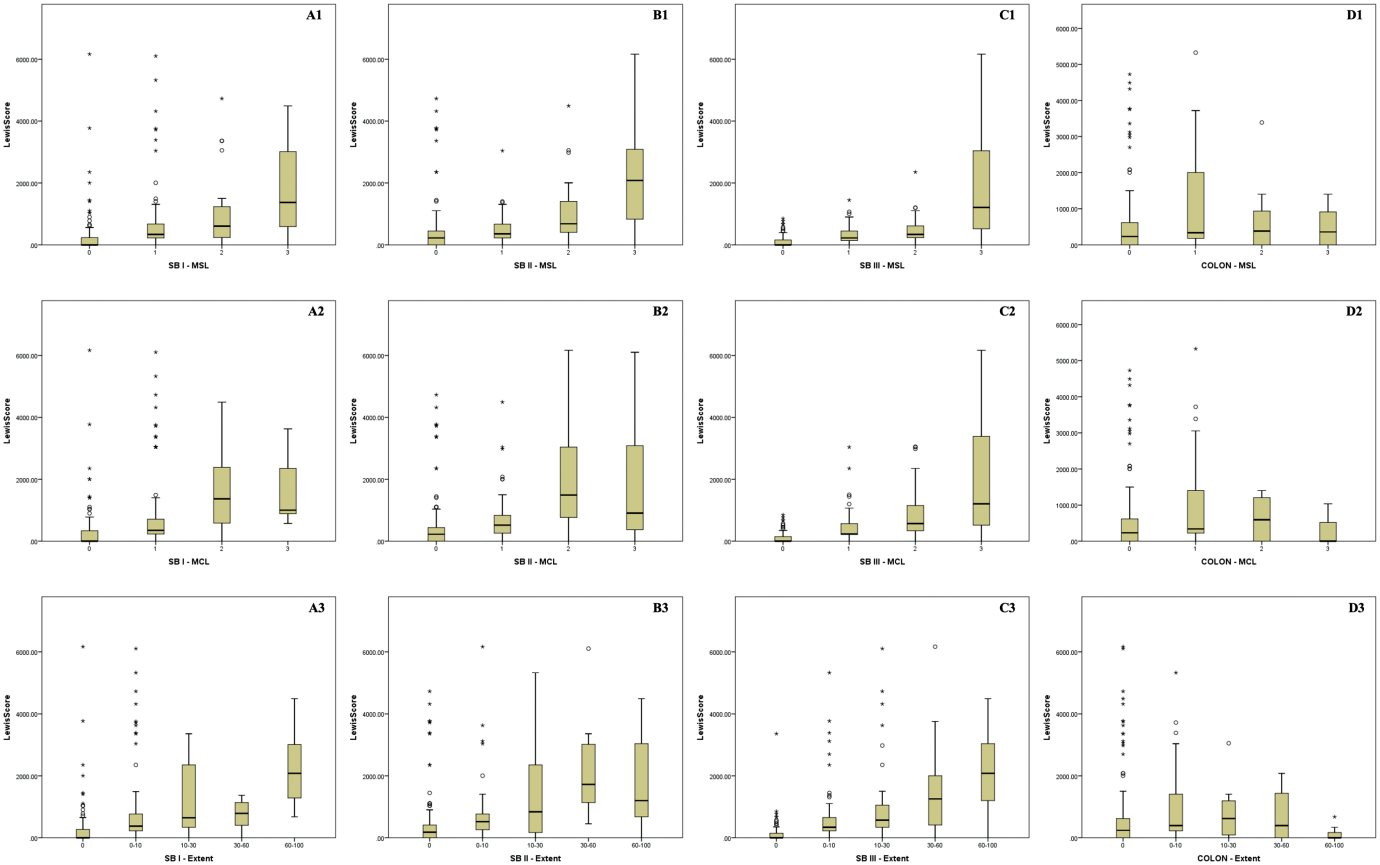
Median Lewis score value of the population stratified according to GI segments and characteristics. A, First SB tertile; (B) Second SB tertile; (C) Third SB tertile; (D) colon; (1) MSL—most severe lesion; (2) MCL—most common lesion; (3) extent of the endoscopic features.

To assess the association between clinical scores and biomarkers with the presence of significant endoscopic lesions at PCE, the study cohort was analyzed for MSL and MCL scores (none or mild vs moderate to severe) and for disease extent (< or >30%). Table 3 summarizes the differences in demographic characteristics, clinical indices, and biomarkers between active and nonactive patients detected by PCE. There was no difference by gender and age, except for the extent where subjects with disease extent >30% were significantly younger than those with <30% (*P* < .001). In terms of clinical scores, there was no difference in CDAI, while PCDAI values were higher in patients with severe or extensive disease (*P* < .05). Hemoglobin and CRP levels differed by severity (*P* < .02), but not by extent, while FCP levels were significantly higher by disease severity and extent (*P* < .04). There was also good agreement between Lewis score values and the PCE scoring system in detecting severe and extensive inflammation (Figure 2). A significant correlation (0.4-0.59) between the Lewis score and the new PCE scoring system was found, with better results particularly in the third tertile (0.6-0.79) and less or no correlation in the colon (Table 4).

**Table 3.** Differences of demographics and noninvasive markers of IBD in the population stratified according to endoscopic features.^*^

	MSL None or Mild (n = 95)	MSL Moderate to Severe (n = 154)	*P*	MCL None or Mild (n = 130)	MCL Moderate to Severe (n = 119)	*P*	Extent < 30% (n = 199)	Extent > 30% (n = 50)	*P*
Age (years)	31.4 (21-47.8)	27.3 (16.9-43.9)	0.119	31 (19.7-45.6)	26 (16.7-43)	0.158	30.8 (19.1-47.4)	17 (12.7-28.9)	<0.001
Sex (F) (n, %)	39 (41.1%)	58 (37.7%)	0.344	51 (39.2%)	46 (38.7%)	0.515	81 (40.7%)	16 (32%)	0.331
CDAI (n = 132, 83%)	140 (83-228)	186 (107-259)	0.172	158 (93-226)	181 (99-265)	0.370	168 (97-246)	224 (102-299)	0.256
PCDAI (n = 82, 91.1%)	8 (0-25)	25 (12.5-40)	0.002	10 (2.5-25)	30 (15-50)	<0.001	13.8 (5-33.5)	25 (12.5-41)	0.046
Hb (g/dL) (n = 190, 76.3%)	14 (12.9-14.6)	12.8 (10.9-13.6)	<0.001	13.5 (12.5-14.4)	12.7 (10.9-13.6)	<0.001	13.2 (11.6-14)	13.1 (12-13.6)	0.798
CRP (mg/dL) (n = 211, 84.7%)	0.5 (0.12-2.3)	1.02 (0.49-2.8)	0.010	0.5 (0.15-2.41)	1.06 (0.5-2.81)	0.016	0.75 (0.23-2.37)	1.30 (0.23-3.02)	0.185
Fecal calprotectin (ug/g) (n = 187, 75%)	49 (16-231)	490 (156-989)	<0.001	75 (22-288)	500 (166-981)	<0.001	210 (30-600)	475 (155-735)	0.034
Lewis score (n = 249, 100%)	135 (0-225)	556 (233-1228)	<0.001	137 (0-335)	670 (337-1448)	<0.001	233 (0-562)	1284 (347-3040)	<0.001

**Table 4.** Correlation among Lewis score and endoscopic features.

		Overall		Pediatric		Adult	
		Spearman’s r	*P*	Spearman’s r	*P*	Spearman’s r	*P*
SBFirst segment	MSL	0.532	<0.001*	0.513	<0.001*	0.542	<0.001*
	MCL	0.534	<0.001*	0.503	<0.001*	0.552	<0.001*
	Extent	0.546	<0.001*	0.599	<0.001*	0.498	<0.001*
SB Second segment	MSL	0.447	<0.001*	0.526	<0.001*	0.413	<0.001*
	MCL	0.448	<0.001*	0.498	<0.001*	0.418	<0.001*
	Extent	0.477	<0.001*	0.586	<0.001*	0.412	<0.001*
SB Third segment	MSL	0.630	<0.001*	0.577	<0.001*	0.680	<0.001*
	MCL	0.634	<0.001*	0.505	<0.001*	0.710	<0.001*
	Extent	0.621	<0.001*	0.642	<0.001*	0.638	<0.001*
Colon	MSL	0.090	0.188	0.032	0.777	0.132	0.131
	MCL	0.121	0.076	0.064	0.564	0.062	0.163
	Extent	0.116	0.090	0.077	0.486	0.151	0.086

*Statistically significant.

### Impact on Disease Outcomes and Therapeutic Management

Using the same stratification by MSL, MCL, and disease extent, we analyzed the association between clinical outcomes with the presence of severe or extensive inflammation at PCE in any GI segment. Endoscopic recurrence occurred more frequently with higher values in all 3 capsule endoscopy items, while treatment escalation was only associated with severe disease but not with extent. Conversely, a clinical relapse was observed more frequently only with disease extent >30%. Table 5 summarizes these results. Considering different age groups, treatment escalation in pediatric patients was associated with both disease severity and extent, while in adults no outcome was affected by disease extent (Table 5).

**Table 5.** Differences of disease outcomes in the total population stratified according to endoscopic features.

	MSL None or Mild (n = 95)	MSL Moderate to Severe (n = 154)	*P*	MCL None or Mild (n = 130)	MCL Moderate to Severe (n = 119)	*P*	Extent <30% (n = 199)	Extent >30% (n = 50)	*P*
Treatment escalation	24 (25.3%)	105 (68.2%)	<0.001	39 (30%)	90 (75.6%)	<0.001	97 (48.7%)	32 (64%)	0.108
Hospitalization	1 (1.1%)	2 (1.3%)	0.982	1 (0.8%)	2 (1.7%)	0.669	1 (0.5%)	2 (4%)	0.100
Clinical relapse	6 (6.3%)	22 (14.3%)	0.154	9 (6.9%)	19 (16%)	0.063	16 (8%)	12 (24%)	0.006
Endoscopic relapse	3 (3.2%)	27 (17.5%)	0.002	6 (4.6%)	24 (20.2%)	0.001	13 (6.5%)	17 (34%)	<0.001

Differences of disease outcomes in the pediatric population									
	MSL None or Mild (n = 33)	MSL Moderate to Severe (n = 57)	P	MCL None or Mild (n = 45)	MCL Moderate to Severe (n = 45)	P	Extent < 30% (n = 56)	Extent > 30% (n = 34)	P
Treatment escalation	3 (9.1%)	33 (57.9%)	<0.001	6 (13.3%)	30 (66.7%)	<0.001	15 (26.8%)	21 (61.8%)	0.004
Hospitalization	0 (0%)	2 (3.5%)	0.499	0 (0 %)	2 (4.4%)	0.319	0 (0%)	2 (5.9%)	0.160
Clinical relapse	4 (12.1%)	10 (17.5%)	0.458	5 (11.1%)	9 (20%)	0.063	5 (8.9%)	9 (26.5%)	0.084
Endoscopic relapse	3 (9.1%)	19 (33.3%)	0.035	6 (13.3%)	16 (35.6%)	0.038	7 (12.5%)	15 (44.1%)	0.002

Differences of disease outcomes in the adult population.									
	MSL None or Mild (n = 62)	MSL Moderate to Severe (n = 97)	P	MCL None or Mild (n = 85)	MCL Moderate to Severe (n = 74)	P	Extent < 30% (n = 143)	Extent > 30% (n = 16)	P
Treatment escalation	21 (33.9%)	72 (74.2%)	<0.001	33 (38.8%)	60 (81.1%)	<0.001	82 (57.3%)	11 (68.8%)	0.616
Hospitalization	1 (1.6%)	0 (0%)	0.385	1 (1.2 %)	0 (0%)	0.584	1 0.7%)	0 (0%)	0.516
Clinical relapse	2 (3.2%)	12 (12.4%)	0.067	4 (4.7%)	10 (13.5%)	0.041	11 (7.7%)	3 (18.8%)	0.306
Endoscopic relapse	0 (0%)	8 (8.2%)	0.016	0 (0%)	8 (10.8%)	0.008	6 (4.2%)	2 (12.5%)	0.291

Univariate regression analysis showed that a severe inflammation, as described by both MSL and MCL, was significantly associated with a higher risk of treatment escalation and endoscopic recurrence, while disease extent >30% was associated with a higher risk of endoscopic and clinical recurrence (Table 6).

**Table 6.** Univariate regression analysis among outcomes (dependent variable) and endoscopic features (independent variable).

	MSL Moderate to Severe		MCL Moderate to Severe		Extent >30%	
	Odds ratio (95% CI)	*P*	Odds ratio (95% CI)	*P*	Odds ratio (95% CI)	*P*
Treatment escalation	6.33 (3.54-11.290)	<0.001*	7.17 (4-12.6)	<0.001*	1.77 (0.935-3.38)	0.079
Hospitalization	1.241 (0.11-13.9)	0.861	2.15 (0.19-24)	0.535	8.57 (0.76-96.7)	0.082
Clinical relapse	2.47 (0.96-6.34)	0.060	2.59 (1.12-5.98)	0.026*	3.62 (1.58-8.3)	0.002*
Endoscopic relapse	6.69 (1.96-22.7)	0.002*	5.17 (2-13.18)	0.001*	7.59 (3.35-17.1)	<0.001*

The multivariate analysis revealed several noteworthy findings. In the pediatric population, the extent of the disease was strongly correlated with an elevated risk of clinical recurrence. Additionally, higher values of MCL and MSL were found to be associated with the necessity of treatment escalation in children. Furthermore, a moderate to severe MCL score served as a predictor of endoscopic relapse in this age group. Conversely, among adults, only severe lesions in terms of MCL emerged as a superior predictor for treatment escalation, as emphasized in Table 5. In the comprehensive analysis, moderate to severe MCL and MSL scores exhibited independent associations with treatment escalation. However, when assessing the risk of endoscopic relapse, only MCL demonstrated a significant association. Additionally, a disease extent exceeding 30% was identified as a more reliable indicator of both clinical and endoscopic relapse.

Pan-enteric capsule endoscopy contributed to postexamination therapy switch in 196 of 249 (79%) of the procedures. Of these, 120 (76%) were adults, and 76 (84%) were children. In most cases, treatment intensification/optimization was considered (72% and 83% in adults and children, respectively), while only 3% of adults and 1% of children de-escalated therapy. Fifty-eight investigations were performed in off-therapy patients, specifically 45 (28%) in adults and 13 (14%) in children.

### Technical Details and Capsule Safety

Pan-enteric capsule endoscopy was performed in 101 (41%) patients at the disease onset and/or for staging, in 93 (45%) as follow-up exam to evaluate disease recurrence, and in 55 (14%) to assess response to treatment. Complete pan-enteric examination was achieved in 83% (207 of 249) of subjects, in whom the capsule was excreted before the end of battery life. The excretion rate exhibited a slightly higher percentage in the pediatric population, with 86% compared with 81% in the adult population. However, a complete SB and colon evaluation was obtained in 96% (240 of 249) and 89% (222 of 249), respectively. There was a statistically significant difference between adults and children in median SB transit time (4:56 hours, IQR 3:44-7:01 vs 3:00 hours, IQR 1:47-4:08; *P* < .001), and colonic transit time (6:26 hours, IQR 5:13-7:48 vs 4:26 hours, IQR 2:22-7:04; *P* < .001). Of 22 patients (11%) in whom the colon was incompletely examined (except for colonic stricture), 12 were due to loss of battery power, 3 to loss of capsule signal (1.2%), and 10 to inadequate bowel preparation (4%). Bowel preparation was rated as adequate in 65% of patients, with rates of poor preparation being slightly higher in adults than in children (27.7% vs 23.3%). Patency capsule was systematically performed in adulthood (158 of 159; 99% of procedures), while this test was only considered when investigators identified a risk of possible retention in children (9 of 90; 10% of pediatric procedures). Only 1 case of capsule retention was reported in an adult patient who did not undergo patency but only MRE 6 months prior the capsule exam. Retention was asymptomatic as the patient was in a complete clinical remission and the capsule was retrieved by device-assisted enteroscopy. No case of pediatric retention was reported, but all pediatric patients underwent imaging tools (MRE and/or US) prior to capsule examination.

## Discussion

In this multicenter, prospective, observational study, PCE was able to efficiently detect inflammation in all GI segments in both children and adults, thus distinguishing the pediatric phenotype from the adult phenotype. By a complete GI evaluation, pediatric CD appeared more pan-enteric and severe in the colon, thus being associated to different outcomes compared with adults. In the pediatric population, the primary predictor of clinical recurrence was determined to be more extensive disease. Additionally, a correlation was observed between more aggressive disease and the need for treatment escalation or endoscopic recurrence in children. On the other hand, among adults, the presence of severe lesions in any GI segment was associated with treatment escalation, irrespective of the extent of the disease.

These results are important for several reasons. First, this study confirmed that pediatric and adult CD are characterized by different phenotypes, especially for disease extent and severity. In this cohort, pediatric subjects presented a severe colonic disease more frequently than adults. The predominance of colon CD in children has been already reported.^21^ However, it is also true that small bowel is often concurrently involved in pediatric subjects, thus making the disease more pan-enteric compared with adults.^22^ A recent study reported that more than 2 out of 3 of pediatric patients have an extensive pan-enteric phenotype.^28^ Moreover, even adult CDs are often presenting proximal lesions if a proper SB evaluation is performed, thus confirming the idea that the disease needs to be assessed in all GI segments irrespective of the age.^8^ Currently, both pediatric and adult guidelines recommend a SB evaluation at the time of the diagnosis in all CD patients.^5,6^ By using PCE, it seems possible to precisely evaluate the disease extent and severity with a single noninvasive examination in both SB and colon, thus assessing the disease phenotype at any age.

Second, this study also confirmed that mucosal disease activity can be only partially evaluated by clinical indices and biomarkers. The PCDAI and FCP differed by severity and extent, while hemoglobin and CRP only differed by severity. The CDAI did not present a significant difference, but the results on this clinical index can be affected by several missing data. Although higher PCDAI and FCP levels were observed more frequently in severe and extensive lesions, these markers usually fail to establish or measure true disease burden and extent of lesions in the GI tract. Therefore, in clinical practice it is often difficult to properly monitor the disease and accurately quantify mucosal activity using clinical scores or biomarkers. The CALM study showed that tight control of the disease using biomarkers is superior to standard clinical assessment, but better outcomes were only obtained in patients who achieved mucosal healing, confirming that this goal is the main determinant of disease progression.^29^ A recent pediatric study also found that a treat-to-target strategy based on PCE results can increase deep remission rates over time by efficiently guiding therapy according to the presence of mucosal lesions in both the SB and colon. This study suggested that disease monitoring through assessment of endoscopic lesions would be the ideal and preferred method for CD patient management, making disease surveillance more precise and able to truly measure disease burden at any time.^19^ The problem of such endoscopic monitoring could be represented by cost and patient discomfort; however, PCE has shown to be less invasive and better tolerated in all age groups compared with standard colonoscopy and MRE.^30^ A future prospective evaluation should determine the actual increased value of PCE in CD monitoring. It is important to note that ileocolonoscopy with histology continues to play a fundamental role in the initial diagnosis of the disease and for obtaining samples for histological examination and dysplasia surveillance.

Consequently, our results are even important because they confirm that PCE findings have an impact on disease outcomes and therapeutic decisions. Both severity and extent can affect clinical outcomes, with different outcomes by age group. While severity is more often considered a major determinant in disease management, the extent is usually less considered. However, in our pediatric cohort, the disease extent was the main factor associated with a significant risk of clinical recurrence. This finding should raise the question of whether pediatric gastroenterologists should calibrate therapeutic decisions based solely on disease severity. Indeed, a recent international consensus has highlighted the significance of small bowel disease as a risk factor for disease complications.^31^ This consensus supports the understanding that SB involvement is often associated with a higher likelihood of structuring or penetrating disease, which in turn might justify the need for more aggressive treatment strategies. An international capsule study has already shown that the presence of significant inflammation at the SB capsule is superior to that of FCP in predicting the short- and long-term risk of recurrence.^32^

Additionally, when the disease is properly assessed for both severity and extent, investigators usually adjust treatments based on this information.^26^ In this cohort of both pediatric and adult subjects, PCE resulted in a treatment change in approximately 80% of cases. Previous studies have already shown that the more information available, the better investigators’ therapeutic choices will be, allowing treatments to be adjusted based on inflammatory disease burden and location.^33^

In our study, it appears that a more comprehensive or aggressive disease, as determined by the PCE score, may result in intensified treatment or clinical and endoscopic recurrence, regardless of the specific segment affected by the disease. In essence, if the disease is more aggressive, it becomes increasingly challenging to control it over time, even with appropriate treatments and follow-up. One potential approach could involve upstaging the treatment based on an objective evaluation using the PCE, thereby increasing the likelihood of disease control. However, it is important to note that in certain cases, even escalating the treatment may not effectively manage the disease if the lesions and extension are particularly significant. This poses a challenge for IBD experts who cannot accurately predict the efficacy of the therapies employed. Ultimately, what remains evident is that more aggressive diseases typically require a correspondingly aggressive treatment approach, with the hope of impeding disease progression and altering its outcomes.

Pan-enteric capsule endoscopy appeared to be safe, with shorter duration of exams and higher quality of bowel preparation in the pediatric population. This confirms what has already been described that children may have faster peristalsis, which affects completion rate and bowel preparation.^15^ Moreover, given the higher prevalence of the inflammatory phenotype in the pediatric population, the routine utilization of a patency capsule is generally deemed unnecessary due to the lower risk of strictures. Only in scenarios where substantial uncertainties arise should this supplementary test be considered to mitigate the risk of capsule retention. An example of such a scenario is when MRE or US indicates the presence of a nonsevere stricture, but the capsule is needed as a valuable tool to assess mucosal status in patients with predominant small bowel localization. Since MRE and US have a tendency to overestimate stricture presence, performing a patency capsule before considering a standard capsule may be a prudent option in such clinical setting.

Finally, it is essential to recognize that PCE enhances the monitoring of patients with CD by reducing the need for invasive procedures throughout the entire GI tract. This single examination can effectively obviate the necessity for repetitive multiple procedures (ie, colonoscopies, SB capsule, MRE, etc.) over time. However, MRE and US also contribute valuable information at the time of diagnosis and during disease monitoring. It is crucial to acknowledge that imaging techniques, both MRE and US, offer useful insights into the status of the SB intestinal wall with relatively lower accuracy for mucosal lesions. In contrast, capsule endoscopy emerges as a more specific tool for mucosal evaluation. Therefore, a comprehensive approach should be adopted in the assessment of patients with CD, incorporating both imaging techniques and capsule endoscopy, as recommended by recent guidelines.^6^ These modalities provide critical information on 2 pivotal treatment end points—mucosal healing (MH) and transmural healing (TH). While TH appears associated with better long-term outcomes, the latest STRIDE II consensus underscores that MH is currently considered the primary target in CD management.^34^ Hence, relying solely on imaging as a surrogate for small bowel MH may prove insufficient, both at the time of diagnosis and during follow-ups.

This study also has some limitations. First, it is an observational data collection implying a higher risk of potential bias; however, a total of 4 recruiting sites were involved, enrolling a homogeneous and consecutive number of patients, making the study cohort more realistic and results more generalizable.

Secondly, it is worth noting that PCE studies were conducted using varying protocols, as standardization in this area is currently lacking. However, it is important to highlight that all patients underwent assessments by experienced readers who specialize in interpreting PCE in the context of CD. This careful consideration significantly reduced the potential for misinterpretation of the results and allowed for a comprehensive evaluation of the genuine value of PCE in real-world clinical practice. Third, only pediatric patients able to swallow were included in this study; therefore, a subset of the enrollable patients was not included, and our results cannot apply to children who are unable to swallow. Nevertheless, this underscores a practical consideration that needs to be taken into account in everyday clinical practice. Fourth, most of these patients received only 1 assessment by PCE, and longitudinal data are available only for a small subset of patients. Furthermore, not all procedures had available laboratory data. Nonetheless, the authors are continuing this prospective data collection using PCE in CD monitoring, and future results on the longitudinal assessment of both adult and pediatric patients will be reported in a second manuscript. Fifth, we would like to acknowledge that the methodology employed in our outcomes analysis may be considered a limitation. Specifically, we focused on assessing the values of endoscopic findings (MCL, MSL, and extension) solely in the most inflamed segments. However, it is important to highlight that during the data collection phase, the Eliakim’s score, which enables a more comprehensive evaluation of disease severity and extension by incorporating lesions across all 3 small bowel segments and the colon, was not yet available.^35^ While this could potentially impact the statistical analysis, we believe that our methodology tends to underestimate the level of inflammation. Nevertheless, it demonstrates the value of the PCE as a valuable tool even in the absence of standardized measures or validated scoring systems. We recognize the need for future studies to explore the utility of incorporating the Eliakim’s score to further enhance the accuracy and comprehensiveness of disease assessment. Finally, the prevalence of mild to moderate active disease and the low incidence of isolated Crohn’s colitis observed in our cohort underscore the importance of obtaining confirmatory evidence in diverse populations to ensure the generalizability of our study findings.

In conclusion, this study confirms that using PCE to monitor pediatric and adult patients with CD in is feasible and valuable, as demonstrated in this real-world cohort of pediatric and adult patients. By using this method, it is possible to determine extent and location of the disease, which allows the phenotype to be differentiated and therapy to be adjusted accordingly. Disease severity and extent appear to have different importance in predicting clinical outcomes in adults and children, with the former probably being of more importance in adulthood, while the latter in childhood. Future prospective multicenter and longitudinal studies will aim to clarify the potential value of PCE in disease characterization, prediction of clinical outcomes, and therapeutic management.

## Supplementary Data

Supplementary data is available at *Inflammatory Bowel Diseases* online.

izae052_suppl_Supplementary_Tables_1

## Data Availability

Analytic methods and study materials will be made available to other researchers by request.

## Abbreviations:

CE: capsule endoscopy
GI: gastrointestinal
CD: Crohn’s Disease
IBD: inflammatory bowel disease
MRE: magnetic resonance enterography
PCE: pan-enteric capsule endoscopy
PCDAI: pediatric Crohn’s disease activity index
SB: small bowel
MCL: most common lesion
MSL: most severe lesion
