# Supplementary material for: Pan-enteric Capsule Endoscopy to Characterize Crohn’s Disease Phenotypes and Predict Clinical Outcomes in Children and Adults: The Bomiro Study
Source: Inflamm Bowel Dis. 2024 Mar 26;31(3):636–46. doi: 10.1093/ibd/izae052 (PMC12628309; doi:10.1093/ibd/izae052)
Supplement: izae052_suppl_Supplementary_Tables_1 [file izae052_suppl_supplementary_tables_1.docx]

| **Segment** | **MSL** | | **MCL** | | **Extent%** |
| --- | --- | --- | --- | --- | --- |
| **SBI** | 0 = none  1 = mild  2 = moderate  3 = severe | ± S  (stricture) | 0 = none  1 = mild  2 = moderate  3 = severe | ± S  (stricture) | 0  0-10%  10-30%  30-60%  60-100% |
| **SBII** | 0 = none  1 = mild  2 = moderate  3 = severe | ± S  (stricture) | 0 = none  1 = mild  2 = moderate  3 = severe | ± S  (stricture) | 0  0-10%  10-30%  30-60%  60-100% |
| **SBIII** | 0 = none  1 = mild  2 = moderate  3 = severe | ± S  (stricture) | 0 = none  1 = mild  2 = moderate  3 = severe | ± S  (stricture) | 0  0-10%  10-30%  30-60%  60-100% |
| **Colon** | 0 = none  1 = mild  2 = moderate  3 = severe | ± S  (stricture) | 0 = none  1 = mild  2 = moderate  3 = severe | ± S  (stricture) | 0  0-10%  10-30%  30-60%  60-100% |

**Supplementary Table 1.** PCE scoring system.

MSL= Most Severe Lesion; MCL= Most Common Lesion; SB= Small Bowel
